# 1695. Epidemiology of Positive Blood Cultures with Negative BioFire^®^ Blood Culture Identification Panel Results at a Pediatric Center

**DOI:** 10.1093/ofid/ofad500.1528

**Published:** 2023-11-27

**Authors:** Pratik A Patel, Miguel A Locsin, Katie Liu, Preeti Jaggi, Mark D Gonzalez

**Affiliations:** Emory University School of Medicine, Alpharetta, GA; Emory University School of Medicine, Alpharetta, GA; Emory University School of Medicine, Alpharetta, GA; Emory University, Atlanta, GA; Children's Healthcare of Atlanta, Atlanta, Georgia

## Abstract

**Background:**

The BioFire^®^ Blood Culture Identification (BCID) Panel is a rapid multiplex PCR assay designed to identify common bloodstream infection (BSI) organisms from positive blood cultures (Figure 1). Although a positive BCID result can identify and often guide pathogen-directed treatment, a negative result may lead to uncertainty. Therefore, we aimed to describe the organisms isolated from cultures with negative BCID tests in pediatric patients.

Figure 1

**Figure:**
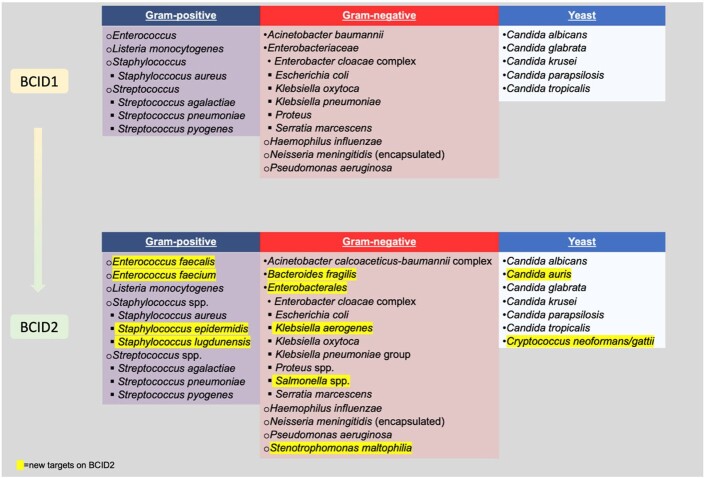

Targets on the BioFire Blood Culture Identification Panels 1 vs. 2

**Methods:**

In this retrospective study of children (≤ 21 years) in our health system, we extracted all BCID tests performed 01/01/2018 to 03/12/2023 from the electronic medical record (EMR). We excluded positive BCID tests and from the negative tests, excluded duplicates, those with no growth and isolated Gram-positive rod Gram stain. Demographic, clinical, and culture data were abstracted from the EMR. Host status was categorized as previously healthy (PH), chronic condition (CC), and immunocompromised (IC). Descriptive statistics were used to summarize the data.

**Results:**

Of the 4049 BCID tests (3123 BCID1, 926 BCID2) performed, 387 resulted negative (327 BCID1 [10.4%], 61 BCID2 [6.6%]). After applying exclusion criteria, 351 negative BCID tests (296 BCID1, 55 BCID2) from 325 patients were included in the dataset. Among the 351 negative tests, 81 (23.1%) were in PH hosts, 151 (43.0%) in CC hosts, and 119 (33.9%) in IC hosts. The majority (n=208, 59.3%) of negative test results were from cultures drawn peripherally. Eighteen tests (5.1%) resulted in polymicrobial growth, and 156 (44.4%) were not treated as BSIs by the clinician (Table 1).

After the BCID negative result, the median delay in organism identification using conventional methods was 28.9 hours. The 351 cultures from negative BCID tests yielded 372 organisms with the most common *Micrococcus* spp. (Figure 2).

Table 1

**Figure:**
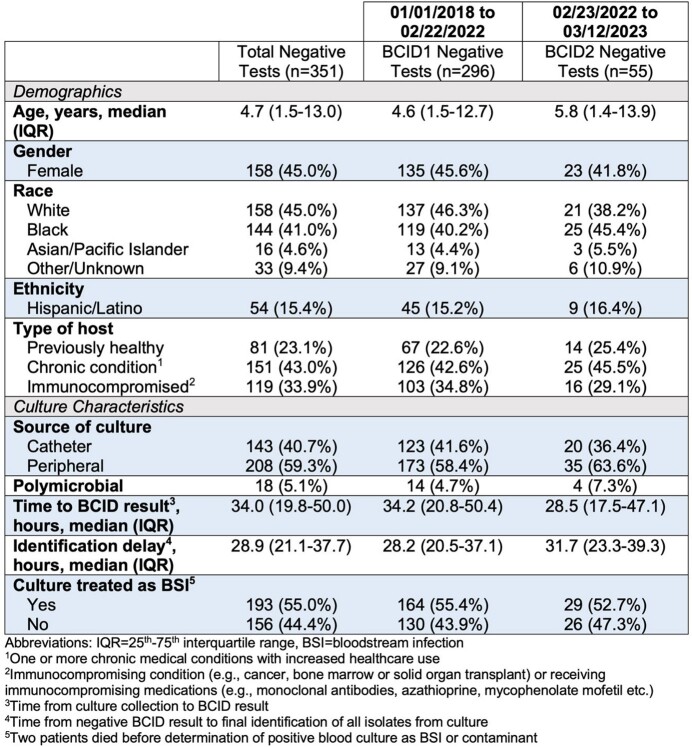

Demographic and culture characteristics of patients with negative BioFire BCID test results: a comparison between BCID1 and BCID2

Figure 2

**Figure:**
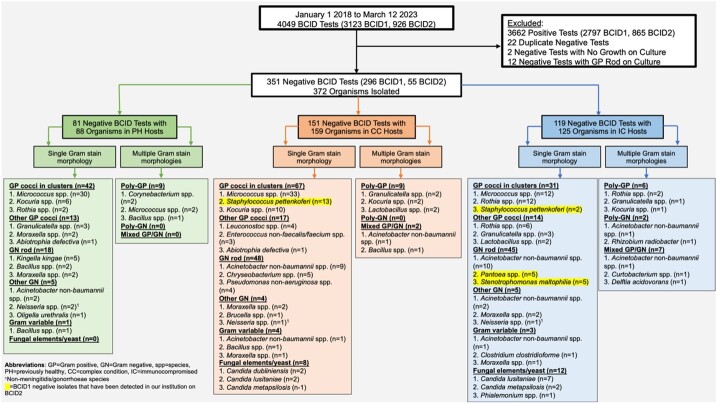

Top 3 organisms isolated from positive cultures with negative BioFire BCID tests, stratified by patient host status and reported Gram stain

**Conclusion:**

In this large pediatric cohort, the BioFire^®^ BCID1 was negative in 10.4% of tests, decreasing to 6.6% with BCID2. We provide clinicians with a useful resource of organisms isolated from cultures with negative BCID results based on underlying host status. With an identification delay of > 24 hours for the majority of BCID negative tests and many not ultimately treated as BSIs, the impact of negative BCID results on antimicrobial stewardship efforts remains an area of future study.

**Disclosures:**

**Pratik A. Patel, MD**, Cardinal Health Inc: Advisor/Consultant

